# The Connection between Bayesian Inference and Information Theory for Model Selection, Information Gain and Experimental Design

**DOI:** 10.3390/e21111081

**Published:** 2019-11-04

**Authors:** Sergey Oladyshkin, Wolfgang Nowak

**Affiliations:** Department of Stochastic Simulation and Safety Research for Hydrosystems, Institute for Modelling Hydraulic and Environmental Systems/SC SimTech, University of Stuttgart, Pfaffenwaldring 5a, 70569 Stuttgart, Germany

**Keywords:** model evidence, entropy, model selection, information entropy, Bayesian experimental design, Kullback–Leibler divergence, Markov chain Monte Carlo, Monte Carlo

## Abstract

We show a link between Bayesian inference and information theory that is useful for model selection, assessment of information entropy and experimental design. We align Bayesian model evidence (BME) with relative entropy and cross entropy in order to simplify computations using prior-based (Monte Carlo) or posterior-based (Markov chain Monte Carlo) BME estimates. On the one hand, we demonstrate how Bayesian model selection can profit from information theory to estimate BME values via posterior-based techniques. Hence, we use various assumptions including relations to several information criteria. On the other hand, we demonstrate how relative entropy can profit from BME to assess information entropy during Bayesian updating and to assess utility in Bayesian experimental design. Specifically, we emphasize that relative entropy can be computed avoiding unnecessary multidimensional integration from both prior and posterior-based sampling techniques. Prior-based computation does not require any assumptions, however posterior-based estimates require at least one assumption. We illustrate the performance of the discussed estimates of BME, information entropy and experiment utility using a transparent, non-linear example. The multivariate Gaussian posterior estimate includes least assumptions and shows the best performance for BME estimation, information entropy and experiment utility from posterior-based sampling.

## 1. Introduction

Probability theory and stochastic analysis provide powerful tools for model selection, parameter inference, data assimilation and experimental design. Bayesian inference is a branch of classical probability theory [1] that offers a stochastic framework for inverse modelling and for assessing the remaining uncertainty in model parameters and prediction [2]. Bayesian principles can be approached via prior-based sampling approaches such as Monte Carlo [3] or via posterior-based sampling approaches as Markov chain Monte Carlo (MCMC: [4]). A number of different approaches for model comparison and selection are available in the literature. Typically, some trade-off between the model’s skill and its degree of complexity is sought for in order to identify a model that will yield robust predictions beyond calibration conditions [5]. The Bayesian framework offers the so-called Bayesian model selection or Bayesian model averaging [6]. These two approaches rest on an integral measure of model performance against available observation data, called the Bayesian model evidence (BME, also called marginal likelihood), and use it to provide a relative model ranking or relative model weights [7,8]. Bayesian model selection can also be seen as a special case of decision theory where the model with the largest expected likelihood is rewarded relative to the model with a smaller expected likelihood. Estimating BME could be rigorously achieved via marginalizing the likelihood of data over the prior distribution of the model using prior-based sampling algorithms such as plain Monte Carlo integration. It is well known that such plain Monte Carlo techniques require a large number of model runs, and become computationally very demanding for many applied problems [6,9]. Thermodynamic integration [10], thermodynamic integration combined with parallel tempering [11], nested sampling [12,13], Gaussian mixture importance sampling [14] or employment of surrogates [15] were proposed in the literature to reduce the computational burden of estimating BME. However, surrogates include approximation errors due to the reduced models, so that estimated BME values should incorporate a correction factor that helps to assure a reliable model ranking especially under strong computational time constraints [16]. Posterior-based sampling techniques achieved via Markov chain Monte Carlo are widely used in the literature and seem to be very efficient for Bayesian inference [17]. However, estimating the BME based on posterior samples is known to be biased [18]. This fact poses a very strong limitation for posterior-based estimates of BME required for Bayesian model selection and model averaging. The first attempt to provide BME values from posterior samples were proposed in [19], based on the harmonic mean approximation. Unfortunately, the harmonic mean estimate tends to overestimate BME [20] and it converges to a biased estimate [18]. Gelfand and Dey [21] proposed a simulation-consistent alternative to the harmonic mean estimator, again based on posterior samples. Chib [22] suggests to follow Bayes rule and estimate BME based on a high-density point in the support of the posterior (see also [23]). Computation via the Gelfand–Dey and Chib methods can be found in [24].

Seemingly unrelated at first sight, information theory grew up in the 1940s [25,26,27,28] from classical probability theory [1]. Information entropy [26] and cross entropy [25,29] were widely used in the literature to measure expected uncertainty and information (see e.g., [30,31]). Relative entropy, also called Kullback–Leibler divergence [27], measures the difference between two probability distributions. All mentioned entropies are widely used for model section [32,33,34], optimal design of experiments [35,36,37,38] and as well for machine learning [39,40,41]. Bayes’s rule was shown to be informationally efficient, and Bayes’s theorem has been linked to maximum-entropy concepts in [42]. A recent review on entropy, information theory, information entropy and Bayesian inference can be found in the paper [43] by Mohammad-Djafari. However, according to definition, all entropies require estimation of a multidimensional integral. To avoid that integral in applications, various approximations such as the Akaike information criterion [44], a second-order bias correction of the Akaike information criterion [45], the Kashyap information criterion [46], the Bayesian information criterion [8], and many others were developed.

In these criteria, in model selection and in experimental design, information theory and Bayesian statistics encounter each other. Usually, these criteria rest on strong assumptions about the models under consideration that are rarely met in practice, especially when nonlinear models are involved [6]. When applied although these conditions are not met, only parts of the available information about a model’s skill and complexity are used (e.g., only the performance at the most likely parameter set), which could yield biased results. Detailed discussion about the various information criteria and also pro-contra arguments for model selection based on Bayesian model evidence or on various information criteria can be found in a recent guiding study [5].

The current paper shows the deep connection between Bayesian inference and information theory in Section 2. This connection can be employed for model selection, assessment of information entropy and experimental design. The scope of the current paper is to align BME with entropies from information theory in order to simplify BME and relative entropy estimations using either prior or posterior-based sampling techniques. Section 3 demonstrates how BME can be estimated via posterior-based MCMC-like techniques using various assumptions. Additionally, Section 3 discuss how BME relates to several information criteria that are known in information theory. Section 4 demonstrates how relative entropy can be computed to assess the information entropy during Bayesian updating and to predict the utility of an experiment during Bayesian experimental design. We emphasize that the information entropy and the predicted utility of an experiment can be computed avoiding unnecessary multidimensional integration for both prior and posterior-based sampling approaches. Employing prior-based approaches does not require any additional assumptions. However, posterior-based estimates require at least one additional assumption. Multivariate Gaussian posterior estimates similar to the Gelfand and Dey approach [21], include least assumptions among all approximates discussed in Section 3 and hence offer a suitable assessment of BME and information entropy using posterior-based approaches. Section 5 illustrates evidence of convergence for BME, information entropy and experiment utility with our proposed methods using a simple didactic example.

## 2. Bayesian Inference and Information Theory

### 2.1. Bayesian Inference

Bayesian theory offers a statistically rigorous approach to deal with uncertainty during inference, providing probabilistic information on the remaining uncertainty in parameters and predictions while incorporating the available observation data. In the Bayesian framework, initial knowledge of parameters is encoded in a prior probability distribution. After Bayesian parameter inference, one obtains a posterior probability distribution of the parameters, which is more informative than the prior distribution (strictly: as least as informative as). Formally, the posterior parameter distribution $p(\omega |y_{*})$ of *n* uncertain parameters forming the vector of random variables $\omega =\left\{\omega_{1},\ldots ,\omega_{n}\right\}$ from the parameter space Ω is obtained by updating the prior parameter distribution p(ω) in the light of observed data $y_{*}$ (vector) according to Bayes’ Theorem (page 6 in [1]):

(1) $$p\left(\omega |y_{*}\right)=\frac{p\left(y_{*}|\omega \right)p\left(\omega \right)}{p(y_{*})},$$

where the term $p(y_{*}|\omega )$ is the likelihood function that quantifies how well the predictions y(ω) based on specific parameter combinations ω match the observed data $y_{*}$, and the term $p(y_{*})$ is BME.

BME $p(y_{*})$ can bee seen as a normalizing constant for the posterior distribution of the parameters ω and can be obtained from Equation (1) using the property of probability density functions that $\int_{\Omega }p\left(\omega |y_{*}\right)d\omega =1$:

(2) $$\mathrm{BME}\equiv p\left(y_{*}\right)=\int_{\Omega }p\left(y_{*}|\omega \right)p\left(\omega \right)d\omega .$$

BME indicates the quality of the model against the available data and it can be directly estimated [47] from Equation (2) using Monte Carlo (MC) or similar prior-based sampling techniques [48]. Several stochastic computational approaches omit direct computation of the normalizing constant $p(y_{*})$ if only the posterior distribution should be sampled (e.g., rejecting sampling [3] or many MCMC techniques). Markov chain Monte Carlo was shown to be an efficient alternative to Monte-Carlo integration for Bayesian updating [49], by providing samples from the posterior. However, computing BME $p(y_{*})$ is indispensable for Bayesian model selection and Bayesian model averaging frameworks [6] where a relative model ranking based on BME ratios play the core role [7,8]. However, the integral in Equation (2) cannot be estimated directly if only posterior samples are available, unless one is willing to accept a bias in the so-called harmonic mean estimate [18]. This poses a very strong limitation especially if posterior-based techniques such as Markov chain Monte Carlo [1] should be applied to estimate BME [6]. It is annoying that Bayesian updating requires posterior sampling, while estimating BME should use prior sampling; this means that these two tasks need their own samples each. The current paper will demonstrate how the posterior distribution could be employed to estimate BME in a different fashion, and for that we will use the notions of information theory introduced in the next Section 2.2.

### 2.2. Information Theory

In the current Section, we will recall several definitions from Information theory [50] that we will employ to assess information [51] in terms of the probability density functions introduced in Section 2.1. The definitions in the current Section are considered to be well known and hence we refer to the original papers [25,26,27,28] for further interpretation.

Information entropy is a measure of the expected missing information and also can be seen as uncertainty of a random variable ω. According to Shannon [26], the information entropy $H\left[p(\omega |y_{*})\right]$ for a random variable ω with (posterior) parameter distribution $p(\omega |y_{*})$ is defined as the following:

(3) $$H\left[p(\omega |y_{*})\right]=-\int_{\Omega }\ln\left[p(\omega |y_{*})\right]p\left(\omega |y_{*}\right)d\omega .$$

The cross entropy [25,29] between two probability distributions is a measure of the expected information that is required to get from one distribution to another. Therefore, the cross entropy $H\left[p\left(\omega |y_{*}\right),p\left(\omega \right)\right]$ is a measure of the expected missing information required to obtain the posterior $p(\omega |y_{*})$ from the prior p(ω):

(4) $$H\left[p\left(\omega |y_{*}\right),p\left(\omega \right)\right]=-\int_{\Omega }\ln\left[p(\omega )\right]p\left(\omega |y_{*}\right)d\omega .$$

Additionally, similar to Equation (4) we will introduce for the further use a non-normalized cross entropy $\hat{H}\left[p\left(\omega |y_{*}\right),p\left(y_{*}|\omega \right)\right]$ that estimates non-normalized expected missing information required to obtain the posterior $p(\omega |y_{*})$ from the likelihood $p(y_{*}|\omega )$:

(5) $$\hat{H}\left[p\left(\omega |y_{*}\right),p\left(y_{*}|\omega \right)\right]=-\int_{\Omega }\ln\left[p(y_{*}|\omega )\right]p\left(\omega |y_{*}\right)d\omega .$$

This cross entropy $\hat{H}\left[p\left(\omega |y_{*}\right),p\left(y_{*}|\omega \right)\right]$ is non-normalized because the likelihood $p(y_{*}|\omega )$ is a proper probability density in the space of measurement data $y_{*}$ only, but not a proper probability density in the space of modelling parameters ω. Therefore, we will introduce the normalized cross entropy $H\left[p\left(\omega |y_{*}\right),p\left(y_{*}|\omega \right)\right]$ that relies on the likelihood normalized by the probability of data $p(y_{*})$:

(6) $$H\left[p\left(\omega |y_{*}\right),p\left(y_{*}|\omega \right)\right]=-\int_{\Omega }\ln\left[\frac{p(y_{*}|\omega )}{p(y_{*})}\right]p\left(\omega |y_{*}\right)d\omega =\ln\mathrm{BME}+\hat{H}\left[p\left(\omega |y_{*}\right),p\left(y_{*}|\omega \right)\right].$$

Another well-known characteristic of information is relative entropy, also called Kullback–Leibler divergence $D_{\mathrm{KL}}$. It measures the difference between two probability distributions [27] in the Bayesian context. The relative entropy $D_{\mathrm{KL}}\left[p\left(\omega |y_{*}\right),p\left(\omega \right)\right]$ measures the so-called information geometry in moving from the prior p(ω) to posterior $p(\omega |y_{*})$ or information lost when p(ω) is used to approximate $p(\omega |y_{*})$:

(7) $$D_{\mathrm{KL}}\left[p\left(\omega |y_{*}\right),p\left(\omega \right)\right]=\int_{\Omega }\ln\left[\frac{p(\omega |y_{*})}{p(\omega )}\right]p\left(\omega |y_{*}\right)d\omega ,$$

or, using the definitions in Equations (3) and (4):

(8) $$D_{\mathrm{KL}}\left[p\left(\omega |y_{*}\right),p\left(\omega \right)\right]=H\left[p\left(\omega |y_{*}\right),p\left(\omega \right)\right]-H\left[p(\omega |y_{*})\right].$$

Relative entropy $D_{\mathrm{KL}}\left[p\left(\omega |y_{*}\right),p\left(\omega \right)\right]$ is usually employed for Bayesian experimental design [35] where expected (marginalized) utility should be maximized [37]. Estimating the relative entropy in Equation (7) requires a multidimensional integration that is often infeasible for applied problems. The link between Bayesian inference and information theory in the current paper will demonstrate how to avoid this multidimensional integration. We would like to remind readers that $D_{\mathrm{KL}}\left[p\left(\omega \right),p\left(\omega |y_{*}\right)\right]\neq D_{\mathrm{KL}}\left[p\left(\omega |y_{*}\right),p\left(\omega \right)\right]$ unless $p\left(\omega \right)=p\left(\omega |y_{*}\right)$, and hence relative entropy cannot be considered a true measure of distance [52].

### 2.3. From Bayesian Inference to Information Theory

We will re-formulate Bayes’ Theorem in Equation (1) to create a useful link between Bayesian Inference and Information Theory. To do so, we will divide Equation (1) by the prior distribution p(ω) and then take the natural logarithm on both sides of the equation:

(9) $$\ln\left[\frac{p(\omega |y_{*})}{p(\omega )}\right]=\ln\left[\frac{p(y_{*}|\omega )}{p(y_{*})}\right].$$

Multiplying Equation (9) by the posterior distribution $p(\omega |y_{*})$ and taking the integral over the parameter space Ω, Bayes’ Theorem becomes:

(10) $$\int_{\Omega }\ln\left[\frac{p(\omega |y_{*})}{p(\omega )}\right]p\left(\omega |y_{*}\right)d\omega =\int_{\Omega }\ln\left[\frac{p(y_{*}|\omega )}{p(y_{*})}\right]p\left(\omega |y_{*}\right)d\omega ,$$

or, decomposing the integral in the right-hand side of Equation (10), we obtain:

(11) $$\int_{\Omega }\ln\left[\frac{p(\omega |y_{*})}{p(\omega )}\right]p\left(\omega |y_{*}\right)d\omega =\int_{\Omega }\ln\left[p(y_{*}|\omega )\right]p\left(\omega |y_{*}\right)d\omega -\int_{\Omega }\ln\left[p(y_{*})\right]p\left(\omega |y_{*}\right)d\omega .$$

Recalling the unit-mass property of probability density functions for $p(\omega |y_{*})$ and then realizing that $\int_{\Omega }\ln\left[p(y_{*})\right]p\left(\omega |y_{*}\right)d\omega =\ln\left[p(y_{*})\right]$, we obtain the following re-formulation of Equation (11):

(12) $$\int_{\Omega }\ln\left[\frac{p(\omega |y_{*})}{p(\omega )}\right]p\left(\omega |y_{*}\right)d\omega =\int_{\Omega }\ln\left[p(y_{*}|\omega )\right]p\left(\omega |y_{*}\right)d\omega -\ln\left[p(y_{*})\right].$$

Equation (12) is a reformulation of Bayes’ Theorem (1) and does not include any simplifications. Hence, without loss of generality, we substitute all necessary definitions from Equations (2), (5) and (7) into Equation (12):

(13) $$D_{\mathrm{KL}}\left[p\left(\omega |y_{*}\right),p\left(\omega \right)\right]=-\ln\mathrm{BME}-\hat{H}\left[p\left(\omega |y_{*}\right),p\left(y_{*}|\omega \right)\right],$$

or, using definition in Equation (6):

(14) $$D_{\mathrm{KL}}\left[p\left(\omega |y_{*}\right),p\left(\omega \right)\right]=-H\left[p\left(\omega |y_{*}\right),p\left(y_{*}|\omega \right)\right].$$

The Equations (13) and (14) are direct consequences of Bayes’ theorem (1) that make use of information theory in the context of Bayesian inference. It is easy to see, that maximizing of the expected relative gain in moving from the prior p(ω) to posterior $p(\omega |y_{*})$ in terms of Kullback–Leibler divergence $D_{\mathrm{KL}}\left[p\left(\omega |y_{*}\right),p\left(\omega \right)\right]$ (Bayesian experimental design [35]) could be obtained if and only if minimizing the missing information required to obtain the posterior $p(\omega |y_{*})$ from the likelihood $p(y_{*}|\omega )$ in terms of cross entropy $H\left[p\left(\omega |y_{*}\right),p\left(y_{*}|\omega \right)\right]$. Overall, decreasing the information loss represented by the cross entropy $H\left[p\left(\omega |y_{*}\right),p\left(y_{*}|\omega \right)\right]$ relies on a compromise between decreasing of the non-normalized cross entropy $\hat{H}\left[p\left(\omega |y_{*}\right),p\left(y_{*}|\omega \right)\right]$ and decreasing BME. From an applied point of view, relative entropy $D_{\mathrm{KL}}\left[p\left(\omega |y_{*}\right),p\left(\omega \right)\right]$ can be used as a model selection criterion. It assigns the highest score to the model that assures a very informative distribution of likelihood compared to the true probability distribution. That means, one would not necessarily select the model with the highest expected value of likelihood (as in traditional BME-based model selection), but the model that provides an overall distribution of normalized likelihood $p(y_{*}|\omega )/\mathrm{BME}$ (including tails, etc.) most similar to the unknown true probability distribution.

The link between Bayesian inference and information theory in Equation (13) can be extended towards assessing overall expected missing information in terms of the information entropy $H\left[p(\omega |y_{*})\right]$ using the definitions in Equations (3), (4) and (8):

(15) $$H\left[p(\omega |y_{*})\right]=\ln\mathrm{BME}+\hat{H}\left[p\left(\omega |y_{*}\right),p\left(y_{*}|\omega \right)\right]+H\left[p\left(\omega |y_{*}\right),p\left(\omega \right)\right],$$

or, using the definition in Equation (6):

(16) $$H\left[p(\omega |y_{*})\right]=H\left[p\left(\omega |y_{*}\right),p\left(y_{*}|\omega \right)\right]+H\left[p\left(\omega |y_{*}\right),p\left(\omega \right)\right].$$

Minimizing the expected information loss in terms of the information entropy $H\left[p(\omega |y_{*})\right]$ has been suggested to identify the best fitting model [44] for model selection and is often used in machine learning. Equation (16) demonstrates that the total expected missing information quantified by $H\left[p(\omega |y_{*})\right]$ corresponds to aggregation of expected missing information required to obtain the posterior $p(\omega |y_{*})$ from the likelihood $p(y_{*}|\omega )$ and the posterior $p(\omega |y_{*})$ from the prior p(ω).

From a model selection point of view, the information entropy $H\left[p(\omega |y_{*})\right]$ prioritises not only the model which offers the most likely prediction of the unknown true probability distribution using the available data (similar to Equation (14)), but as well the model that includes the most informative prior. The last component encourages modellers to provide very meaningful priors and check how close the suggested priors could be to the unknown true probability distributions. Therefore, obtaining an informative likelihood is only one component in information entropy-based model selection. Moreover, Equation (15) explicitly states that minimizing the expected information loss represented by $H\left[p(\omega |y_{*})\right]$ serves not the same purpose as maximizing BME that is often used in the traditional Bayesian model selection framework. Therefore, a proper objective of model selection should be exactly defined (see review [5]).

## 3. Bayesian Model Selection

As already pointed out in Section 2.1, BME is often used for model selection in order to identify the most suitable model among a set of competing models or to rank the competing models. In the Bayesian model selection framework, the prior distribution $p(\omega |M_{k})$, the likelihood function $p(y_{o}|M_{k},\omega )$, the posterior distribution $p(\omega |M_{k},y_{*})$ and Bayesian model evidence $p(y_{*}|M_{k})$ are specific to each competing model $M_{k}$. The overall computational procedure per model is identical for all models, and hence the indicator $M_{k}$ will be omitted in the following. Additionally, Equation (2) shows that BME is equal to the expected value $E_{p(\omega )}$ of the likelihood $p(y_{*}|\omega )$ over the prior p(ω):

(17) $${\mathrm{BME}}_{\mathrm{prior}}=E_{p(\omega )}\left(p(y_{*}|\omega )\right).$$

It is well known that prior-based integration approaches require high computational costs to estimate BME. Therefore, computing BME values from posterior-based sampling, while avoiding the so-called harmonic mean estimator (see Section 2.1), will be very valuable for the applied tasks. To do so, we will employ the newly developed link between Bayesian inference and information theory in Equations (13) and (15). It offers a pathway to estimate BME values from samples representing the posterior distribution $p(\omega |y_{*})$. To do so, we will re-formulate Equation (15) to obtain the following posterior-based representation of BME:

(18) $$\ln{\mathrm{BME}}_{\mathrm{post}}=E_{p(\omega |y_{*})}\left(\ln\left[p(y_{*}|\omega )\right]\right)+E_{p(\omega |y_{*})}\left(\ln\left[p(\omega )\right]\right)-E_{p(\omega |y_{*})}\left(\ln\left[p(\omega |y_{*})\right]\right).$$

Apparently, Equation (18) is based on posterior expectations only. The first term $E_{p(\omega |y_{*})}\left(\ln\left[p(y_{*}|\omega )\right]\right)$ in Equation (18) estimates the non-normalized cross entropy $\hat{H}\left[p\left(\omega |y_{*}\right),p\left(y_{*}|\omega \right)\right]$ from Equation (6). It can be directly computed using posterior samples and the corresponding log-likelihoods. Thus it does not require any knowledge about posterior density values $p(\omega |y_{*})$ or normalization of the posterior. Similarly, the second term $E_{p(\omega |y_{*})}\left(\ln\left[p(\omega )\right]\right)$ in Equation (18) estimates the cross entropy $H\left[p\left(\omega |y_{*}\right),p\left(\omega \right)\right]$ from Equation (4) and can directly be computed by evaluating prior density values of posterior samples. However, the third term $E_{p(\omega |y_{*})}\left(\ln\left[p(\omega_{i}|y_{*})\right]\right)$ responsible for the posterior information entropy $H\left[p(\omega |y_{*})\right]$ poses a serious computational challenge because posterior density values $p(\omega |y_{*})$ are unknown and a posterior sample is available only. Thus, the third term in Equation (18) includes the entire hardness of BME estimation in concentrated form. The upcoming Section 3.1, Section 3.2, Section 3.3, Section 3.4, Section 3.5 and Section 3.6 will offer several options to approximate this term. Additionally, we will demonstrate how the resulting approximations of BME relate to several information criteria known from information theory.

### 3.1. Model Evidence via Posterior Density Estimates

The first possible approximation of the posterior information entropy $H\left[p(\omega |y_{*})\right]$ is to use a density estimate $\tilde{p}\left(\omega |y_{*}\right)$ of the posterior distribution $p(\omega |y_{*})$ based on posterior samples, e.g., via Kernel density estimation [53] and other approaches [54]. Using such an estimate, Equation (18) will lead to the following BME approximation:

(19) $$\ln{\mathrm{BME}}_{\mathrm{post}}^{\mathrm{PDE}}\approx E_{p(\omega |y_{*})}\left(\ln\left[p(y_{*}|\omega )\right]\right)+E_{p(\omega |y_{*})}\left(\ln\left[p(\omega )\right]\right)-E_{p(\omega |y_{*})}\left(\ln\left[\tilde{p}\left(\omega |y_{*}\right)\right]\right).$$

The quality of the posterior density estimate (PDE) $\tilde{p}\left(\omega |y_{*}\right)$ depends on the choice of approach and related assumptions [55]. However, it is well known that any density estimation is extremely computationally demanding and unfeasible for high-dimensional problems (due to the curse of dimensionality). For that reason, we do not recommend to use Equation (19) for applications with many uncertain parameters, but we still use it for demonstration purposes in Section 5.

### 3.2. Model Evidence via Dirac at the Maximum a Posteriori Estimate

Once could assume that the posterior distribution $p(\omega |y_{*})$ has the form of Dirac function. i.e., the only relevant posterior density is concentrated precisely at a single peak. Then, we can approximate $E_{p(\omega |y_{*})}\left(\ln\left[p(\omega |y_{*})\right]\right)$ in Equation (18) via the maximum a posteriori (MAP) parameter set $\omega_{MAP}$:

(20) $$\ln{\mathrm{BME}}_{\mathrm{post}}^{\mathrm{MAP}}\approx E_{p(\omega |y_{*})}\left(\ln\left[p(y_{*}|\omega )\right]\right)+E_{p(\omega |y_{*})}\left(\ln\left[p(\omega )\right]\right)-\ln\left[p\left(\omega_{MAP}\right)|y_{*}\right].$$

We consider this MAP approximation very simplified and suggest to use it as a very rough guess only.

### 3.3. Model Evidence via the Chib Estimate

Even more drastically then in Section 3.2, Chib [22] suggested to reduce computation of BME values to a single point estimate. For estimation efficiency, the point is generally taken to have high probability density (e.g., $\omega_{MAP}$) in the support of the posterior [23]):

(21) $$\ln{\mathrm{BME}}_{\mathrm{post}}^{\mathrm{CHIB}}=\ln\left[p(y_{*}|\omega_{MAP})\right]+\ln\left[p(\omega_{MAP})\right]-\ln\left[p\left(\omega_{MAP}\right)|y_{*}\right].$$

It is easy to see that Chib’s approach is a consistent point estimate of Equation (18). As a direct consequence of Equation (1), it could be exact if we had a perfect posterior density estimate at the MAP for the third term. Its advantage is that it is simple to compute.

### 3.4. Model Evidence via the Akaike Information Criterion

The MAP approximation from Section 3.2 could be extended while employing the Akaike information criterion (AIC) [44]:

(22) $$\mathrm{AIC}=-2\ln\left[p(\omega_{MAP}|y_{*})\right]+2n.$$

Originally, the AIC employed the maximum likelihood estimator, but is often modified to use a MAP estimator $\omega_{MAP}$. The AIC is well discussed in the literature, e.g., [5]. However, the original paper of Akaike [44] used relative entropy, and dropping out the entropy of data (which is an irrelevant constant during model ranking) focuses the AIC to approximate the cross entropy $H\left[p(\omega |y_{*})\right]$ of the calibrated model against the data (page 718: Section IV, paragraph 1 in [44]). Moreover, the original Akaike paper includes an assumption that the calibrated model can hit the real distribution of the observed data (page 718: Section IV, paragraph 3 in [44]). Therefore, indeed, the AIC tends to approximate the entropy $H\left[p(\omega |y_{*})\right]$ in Equation (3) through the relation $\frac{1}{2n}\mathrm{AIC}$ (page 719: Section V, paragraph 1 in [44]). This finding could be directly employed to approximate the last term in Equation (18) as:

(23) $$E_{p(\omega |y_{*})}\left(\ln\left[p(\omega |y_{*})\right]\right)\approx -\frac{1}{2n}\mathrm{AIC}.$$

Hence, substituting the approximation in Equation (23) into Equation (18), BME can be estimated via the AIC as:

(24) $$\ln{\mathrm{BME}}_{\mathrm{post}}^{\mathrm{AIC}}\approx E_{p(\omega |y_{*})}\left(\ln\left[p(y_{*}|\omega )\right]\right)+E_{p(\omega |y_{*})}\left(\ln\left[p(\omega )\right]\right)-\frac{1}{n}\ln\left[p(\omega_{MAP}|y_{*})\right]+1,$$

The paper [45] suggests to account for a second-order bias correction for a limited sample size *s* (length of vector $y_{*}$) and extends the Akaike information criterion to the following from:

(25) $$\mathrm{AICc}=\mathrm{AIC}+\frac{2n(n+1)}{s-n-1}.$$

Therefore, using the correction in the Equation (25), the posterior-based BME estimate in Equation (18) can be written as:

(26) $$\ln{\mathrm{BME}}_{\mathrm{post}}^{\mathrm{AICc}}\approx E_{p(\omega |y_{*})}\left(\ln\left[p(y_{*}|\omega )\right]\right)+E_{p(\omega |y_{*})}\left(\ln\left[p(\omega )\right]\right)-\frac{1}{n}\ln\left[p(\omega_{MAP}|y_{*})\right]+\frac{s}{s-n-1}.$$

It is easy to see that the relations (24) and (26) inherit the maximum a posteriori estimation from the Akaike approach and hence are only approximations. However, we expect that the AIC and AICc-based BME estimate will be superior to a very simplistic MAP estimate as in Equation (20).

### 3.5. Model Evidence via Multivariate Gaussian Posterior Estimates

Assuming that the posterior distribution $p(\omega |y_{*})$ is a multivariate Gaussian (MG) distribution, the information entropy $H\left[p(\omega |y_{*})\right]$ in Equation (3) can be approximated analytically [56,57]:

(27) $$H\left[p(\omega |y_{*})\right]\approx \frac{1}{2}\ln\left[{\left(2\pi e\right)}^{n}\left|C\right|\right].$$

where C is the posterior (co)variance matrix and *n* is the number of uncertain parameters.

Substituting the multivariate Gaussian approximation in Equation (27) into Equation (18), we obtain the following BME estimate:

(28) $$\ln{\mathrm{BME}}_{\mathrm{post}}^{\mathrm{MG}}\approx E_{p(\omega |y_{*})}\left(\ln\left[p(y_{*}|\omega )\right]\right)+E_{p(\omega |y_{*})}\left(\ln\left[p(\omega )\right]\right)+\frac{1}{2}\ln\left[{\left(2\pi e\right)}^{n}\left|C\right|\right].$$

This BME estimate can be directly calculated from posterior-based approaches such as MCMC. Equation (28) provides a superior approximation of BME values in comparison to the various versions of maximum a posteriori estimates in Equations (20), (21), (24) and (26), at least for continuous random variables with unimodal and sufficiently symmetric posterior. Additionally, the multivariate Gaussian posterior estimation in Equation (28) overcomes the curse of dimensionality in posterior density estimation (19) and hence will be more efficient for high-dimensional problems.

### 3.6. Model Evidence via the Kashyap Information Criterion Correction

Kashyap [46] introduced the so-called KIC information criterion. It uses the maximum likelihood parameter estimator and is often modified to use the MAP parameter estimator $\omega_{MAP}$ [58]:

(29) $$\mathrm{KIC}=-2\ln\left[p(\omega_{MAP}|y_{*})\right]-2\ln\left[p(\omega_{MAP})\right]-n\ln\left[2\pi \right]-\ln\left[|C|\right],$$

where C is again the posterior (co)variance matrix as in Equation (27).

Taking into consideration that lnBME=−0.5KIC [6], the BME value can be directly estimated as follows:

(30) $$\ln{\mathrm{BME}}_{\mathrm{post}}^{\mathrm{KIC}}\approx \ln\left[p(\omega_{MAP}|y_{*})\right]+\ln\left[p(\omega_{MAP})\right]+\frac{1}{2}\ln\left[{\left(2\pi \right)}^{n}\left|C\right|\right].$$

Apparently, the KIC-based Equation (30) approximates the expectations $E_{p(\omega |y_{*})}\left(\ln\left[p(y_{*}|\omega )\right]\right)$ and $E_{p(\omega |y_{*})}\left(\ln\left[p(\omega )\right]\right)$ in Equation (28) using the MAP estimate $\omega_{MAP}$ and assumes a multivariate Gaussian posterior distribution similarly to Equation (28). However, the KIC in Equation (29) omits the constant −n (i.e., $-\ln{\left(e\right)}^{n}$) and hence the KIC-based Equation (30) is not acting at the proper BME scale. Omitting the constant −n serves the model selection purpose whenever the number of parameters *n* is the same for all competing models. In a more general case, we suggest to use the multivariate Gaussian approximation according to Equation (28) and offer the follow adjustment of the KIC:

(31) $$\mathrm{KICr}=-2\ln\left[p(\omega_{MAP}|y_{*})\right]-2\ln\left[p(\omega_{MAP})\right]-n-n\ln\left[2\pi \right]-\ln\left[|C|\right].$$

Hence, to obtain the corresponding BME value, we re-scale the last term in Equation (30) by the factor exp(n/2) inside the logarithm:

(32) $$\ln{\mathrm{BME}}_{\mathrm{post}}^{\mathrm{KICr}}\approx \ln\left[p(\omega_{MAP}|y_{*})\right]+\ln\left[p(\omega_{MAP})\right]+\frac{1}{2}\ln\left[{\left(2\pi e\right)}^{n}\left|C\right|\right].$$

It is easy to see that the KIC or KICr -based BME estimation in Equations (30) and (32) simplify the multivariate Gaussian BME estimate in Equation (28) towards MAP estimates of the cross entropies $\hat{H}\left[p\left(\omega |y_{*}\right),p\left(y_{*}|\omega \right)\right]$ and $H\left[p\left(\omega |y_{*}\right),p\left(\omega \right)\right]$. From the computational point of view, the KIC-based BME estimates in Equations (32) or (30) require constructing the posterior (co)variance matrix C, e.g., from a posterior sample. The involved averaging over the posterior sample can be performed as well via a posterior sample to directly approximate the expectations $E_{p(\omega |y_{*})}\left(\ln\left[p(y_{*}|\omega )\right]\right)$ and $E_{p(\omega |y_{*})}\left(\ln\left[p(\omega )\right]\right)$ in Equation (28) without any assumptions for MAP estimation. Therefore, the KIC or KICr -based BME estimates include simplifications that are targeted at the calibrate-by-optimization method, when samples for averaging are not available. Due to this simplification, we expect that the plain multi-Gaussian Equation (28) will be superior to Equations (32) or (30).

### 3.7. Model Evidence via the Schwarz Information Criterion Correction

Schwarz [59] introduced the so-called Bayesian information criterion (BIC, also know as Schwarz information criterion). It simplifies Equation (29), retaining a term that varies with the number of parameters and observations, and relies on the maximum likelihood parameter estimator $\omega_{MLE}$:

(33) $$\mathrm{BIC}=-2\ln\left[p(\omega_{MLE}|y_{*})\right]-n\ln s,$$

Therefore, similar to Equation (30), BME values can be directly approximated as follows:

(34) $$\ln{\mathrm{BME}}_{\mathrm{post}}^{\mathrm{BIC}}\approx \ln\left[p(\omega_{MLE}|y_{*})\right]+\frac{n}{2}\ln s.$$

Apparently, BIC-based BME estimation introduces even stronger simplifications in comparison to KIC and KICr. BIC penalizes the dimensionality of the model and can be seen as asymptotical limit of KIC or KICr with growing data set size *s*. Far away from this limit case, it can only be used as rough approximation of BME (see also discussion in [6]).

### 3.8. Model Evidence via the Gelfand and Dey Estimate

Assuming a multivariate Gaussian posterior distribution $p(\omega |y_{*})$ to estimate the information entropy $H\left[p(\omega |y_{*})\right]$ as in Equation (28) is an assumptions also taken by the Gelfand and Dey (GD) estimate [21]. The idea of Gelfand and Dey in the original paper [21] consists of introducing an importance sampling density τ(ω) that could be multivariate Gaussian or t-densities:

(35) $$\ln{\mathrm{BME}}_{\mathrm{post}}^{\mathrm{GD}}\approx \ln E_{p(\omega |y_{*})}^{-1}\left(\frac{\tau (\omega )}{p\left(y_{*}|\omega \right)p\left(\omega \right)}\right).$$

When assuming multivariate Gaussianity of the importance sampling density τ(ω), the Gelfand and Dey approach includes similar assumptions to the multivariate Gaussian estimate in Equation (27). Both approaches have a potential to capture the posterior better than other estimates discussed in the current Section 3. Table 1 offers a brief summary of assumptions behind all estimates discussed in the current Section 3.

## 4. Bayesian View on the Information Gain

The previous Section 3 used the link between Bayesian inference and information theory from Section 2.3 for posterior-based BME estimation. This link could be used for model selection purposes. Additionally, the findings in Section 2.3 express information entropy in terms of relative entropy, and so could be employed for entropy-based model selection and Bayesian experimental design. There is plenty of literature on entropy-based model selection and Bayesian experimental design, which is impossible to summarize here. In the upcoming Section 4.1 and Section 4.2, we will shortly demonstrate how the involved information and relative entropies could be estimated while avoiding the multidimensional integrals.

### 4.1. Information Entropy during Bayesian Inference

The traditional Bayesian model selection framework relies on the BME value only. Information entropy $H\left[p(\omega |y_{*})\right]$ could be used for model selection once the overall goal is to minimize model discrepancy by finding the best-fit model [44] via minimizing $H\left[p(\omega |y_{*})\right]$. A detailed discussion about the various information criteria and also pro-contra arguments for model selection based on the BME or relative entropy can be found in a recent review [5]. However, information entropy $H\left[p(\omega |y_{*})\right]$ in Equation (3) cannot be computed directly from a posterior sample because the posterior density values $p(\omega |y_{*})$ are unknown. To overcome this situation, we will employ the definition of $D_{\mathrm{KL}}\left[p\left(\omega |y_{*}\right),p\left(\omega \right)\right]$ in Equation (8) to express the information entropy $H\left[p(\omega |y_{*})\right]$:

(36) $$H\left[p(\omega |y_{*})\right]=H\left[p\left(\omega |y_{*}\right),p\left(\omega \right)\right]-D_{\mathrm{KL}}\left[p\left(\omega |y_{*}\right),p\left(\omega \right)\right].$$

Substituting the expression for relative entropy $D_{\mathrm{KL}}\left[p\left(\omega |y_{*}\right),p\left(\omega \right)\right]$ from Equation (13) into Equation (36), we obtain:

(37) $$H\left[p(\omega |y_{*})\right]=\ln\mathrm{BME}+H\left[p\left(\omega |y_{*}\right),p\left(\omega \right)\right]+\hat{H}\left[p\left(\omega |y_{*}\right),p\left(y_{*}|\omega \right)\right].$$

Therefore, the prior-based estimate of $H\left[p(\omega |y_{*})\right]$ is equal to the expected log-ratio between BME, the prior and the likelihood:

(38) $$H{\left[p(\omega |y_{*})\right]}_{\mathrm{prior}}=\ln{\mathrm{BME}}_{\mathrm{prior}}-E_{p(\omega |y_{*})}\left(\ln\left[p(\omega )\right]\right)-E_{p(\omega |y_{*})}\left(\ln\left[p(y_{*}|\omega )\right]\right).$$

Equation (38) avoids any assumptions and avoids multidimensional density estimation and integrals in Equation (3). It employs the prior-based estimation of BME values in Equation (17) and posterior-based expectation of prior densities $E_{p(\omega |y_{*})}\left(\ln\left[p(\omega )\right]\right)$ and likelihoods $E_{p(\omega |y_{*})}\left(\ln\left[p(y_{*}|\omega )\right]\right)$. The latter could be obtained using rejecting sampling techniques [3]. Therefore, is not possible to evaluate Equation (38) if only a posterior sample is available. However, employing the assumptions on BME from Section 3, the information entropy can be estimated accordingly:

(39) $$H{\left[p(\omega |y_{*})\right]}_{\mathrm{post}}=\ln{\mathrm{BME}}_{\mathrm{post}}-E_{p(\omega |y_{*})}\left(\ln\left[p(\omega )\right]\right)-E_{p(\omega |y_{*})}\left(\ln\left[p(y_{*}|\omega )\right]\right).$$

Finally, Equation (39) can be evaluated directly from a posterior sample and does not require any additional steps. Obviously, Equation (39) includes an approximation of BME values in comparison to Equation (38). However, the multivariate Gaussian BME estimate in Equation (28) or the Gelfand and Dey estimate in Equation (35) include least assumptions in comparison to the other possible options in Section 3, and so may offer a viable option.

### 4.2. Bayesian Experimental Design and Information Gain

Relative entropy $D_{\mathrm{KL}}\left[p\left(\omega |y_{*}\right),p\left(\omega \right)\right]$ is often employed for Bayesian experimental design where it represents the utility of an experiment outcome in learning about model parameters [35], i.e., the distance between prior p(ω) and posterior $p(\omega |y_{*})$ distributions in Equation (7). By formal maximization of the expected relative entropy $D_{\mathrm{KL}}\left[p\left(\omega |y_{*}\right),p\left(\omega \right)\right]$ [36,37] one can find an optimal design $d_{max}$ from the design space *D*:

(40) $$d_{max}={arg max}_{d\in D}E_{d}\left(D_{\mathrm{KL}}\left[p\left(\omega |y_{*}\right),p\left(\omega \right)\right]\right).$$

Alternatively, using Equation (14), the optimal design problem in Equation (40) can be formulated as:

(41) $$d_{min}={arg min}_{d\in D}E_{d}\left(H\left[p\left(\omega |y_{*}\right),p\left(y_{*}|\omega \right)\right]\right).$$

The main computational challenge in Equation (40) is to estimate the relative entropy $D_{\mathrm{KL}}\left[p\left(\omega |y_{*}\right),p\left(\omega \right)\right]$. For prior-based Bayesian experimental design, we re-formulate the relation between information theory and Bayesian inference in Equation (13) in the following way:

(42) $$D_{\mathrm{KL}}{\left[p\left(\omega |y_{*}\right),p\left(\omega \right)\right]}_{\mathrm{prior}}=E_{p(\omega |y_{*})}\left(\ln\left[p(y_{*}|\omega )\right]\right)-\ln{\mathrm{BME}}_{\mathrm{prior}}.$$

The expression for relative entropy $D_{\mathrm{KL}}\left[p\left(\omega |y_{*}\right),p\left(\omega \right)\right]$ in Equation (42) again avoids any assumptions and avoids computation of the multidimensional density estimate and integral from Equation (7). It employs the prior-based estimation of BME values in Equation (17) and posterior-based expectation of the likelihood $E_{p(\omega |y_{*})}\left(\ln\left[p(y_{*}|\omega )\right]\right)$ that could be obtained using a rejection sampling technique or similar [3].

Similar to Section 4.1, a posterior-based estimation requires an approximation in Equation (8), if the BME value could not be estimated using the prior samples as in Equation (42):

(43) $$D_{\mathrm{KL}}{\left[p\left(\omega |y_{*}\right),p\left(\omega \right)\right]}_{\mathrm{post}}=-E_{p(\omega |y_{*})}\left(\ln\left[p(\omega )\right]\right)+E_{p(\omega |y_{*})}\left(\ln\left[p(\omega |y_{*})\right]\right).$$

Hence, assuming a multivariate Gaussian posterior distribution and employing Equation (27), the relative entropy could be estimated as follows:

(44) $$D_{\mathrm{KL}}{\left[p\left(\omega |y_{*}\right),p\left(\omega \right)\right]}_{\mathrm{post}}=-E_{p(\omega |y_{*})}\left(\ln\left[p(\omega )\right]\right)-\frac{1}{2}\ln\left[{\left(2\pi e\right)}^{n}\left|C\right|\right].$$

Equations (43) or (44) offer a posterior-based approximation of relative entropy $D_{\mathrm{KL}}\left[p\left(\omega |y_{*}\right),p\left(\omega \right)\right]$ for Bayesian experimental design that is similar to the BME estimate in Equation (28). Moreover, the expected value $E_{d}\left(E_{p(\omega |y_{*})}\left(\ln\left[p(\omega )\right]\right)\right)$ for the so-called pre-posterior analysis [60] has the same value for all possible designs *d* and hence it is irrelevant for Bayesian experimental design. Thus, the optimization problem in Equation (40) can be simplified as:

(45) $$d_{max}={arg max}_{d\in D}E_{d}\left(-\frac{n}{2}\ln\left(2\pi e\right)-\frac{1}{2}\ln\left|C\right|\right),$$

where Equation (45) is known in literate as D-optimally [35].

## 5. Model Evidence, Information Entropy and Experiment Utility for a Test Case

**Figure 1:**
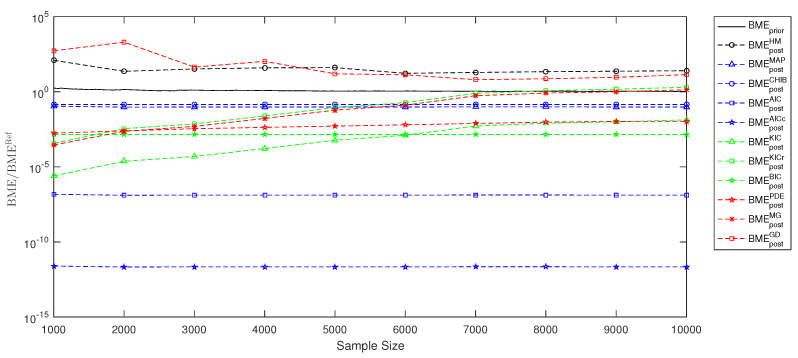
Bayesian model evidence estimation for model selection using Markov chain Monte Carlo and reference Monte Carlo solution.

**Figure 2:**
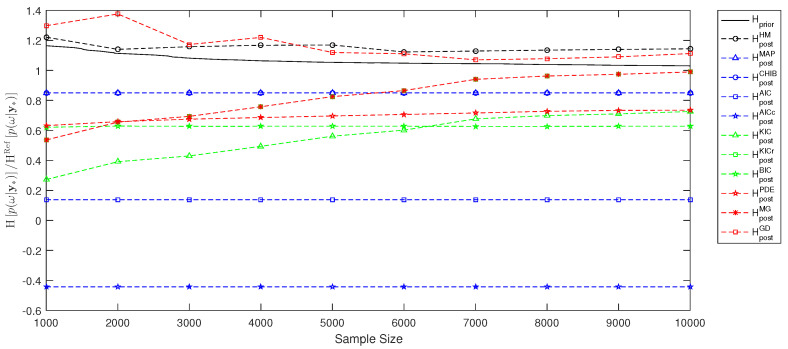
Information entropy estimation for model selection using Markov chain Monte Carlo and reference Monte Carlo solution.

**Figure 3:**
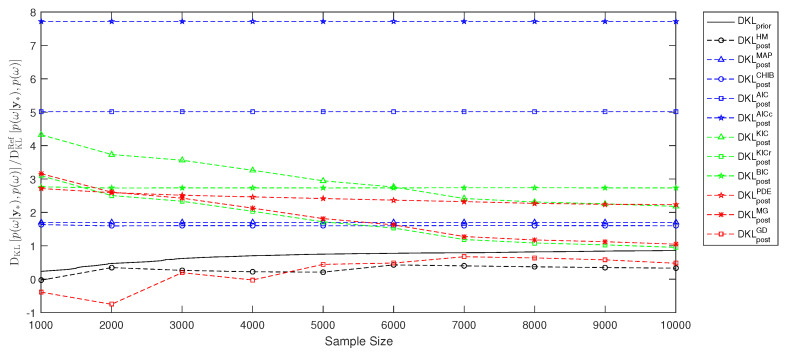
Relative entropy estimate for Bayesian experimental design and model selection using Markov chain Monte Carlo and reference Monte Carlo solution.

In the previous Section 3 and Section 4, we have demonstrated how to employ the link between Bayesian inference and information theory to perform model selection and to assess information entropy for experimental design. The current Section 5 will illustrate the performance of the various estimates from Section 3 and Section 4 using a simple example. This is only a single example out of an infinity of possible applications. These would all differ in prior assumptions, likelihood choices, number of parameters, number of measurement data and degree of non-linearity. Therefore, the resulting Figure 1, Figure 2 and Figure 3 are, of course, problem-specific and can serve as a rough illustration only. The relevant information for the problem-independent properties are the assumptions summarized in Table 1.

### 5.1. Scenario Set Up

Let us introduce a test case scenario in the form of an analytical function that will be used to obtain the necessary quantities of interest for model selection and for assessing information entropy. To make a fair assessment, we will keep in mind the finding in Section 3 and will ensure that the introduced analytical scenario has a non-Gaussian posterior. To do so, we will consider a non-linear analytical function y(ω,t) of ten (n=10) uncertain parameters $\omega =\left\{\omega_{1},\ldots ,\omega_{n}\right\}$:

(46) $$y\left(\omega ,t\right)={\left(\omega_{1}^{2}+\omega_{2}-1\right)}^{2}+\omega_{1}^{2}+0.1\omega_{1}\exp\left(\omega_{2}\right)-2\omega_{1}\sqrt{0.5t}+1+\sum_{i=2}^{n}\frac{\omega_{i}^{3}}{i},$$

where the prior parameter distribution p(ω) is considered to be independent and uniform with $\omega_{i}∼U\left(-5,5\right)$ for $i=\bar{1,10}$.

The prior assumptions on the parameters will be updated using observation data $y_{*}$. For the considered test scenario, we generate ten synthetic observed data values $y_{*}=y\left(\omega ,t_{k}\right)$ with $t_{k}=\left(k-1\right)/9$ and $k=\bar{1,10}$ that correspond to the parameter set $\omega_{i}=0$∀i. To describe how well the predictions y(ω,t) in Equation (46) match the synthetic observed data $y_{*}$, we use the following likelihood function $p(y_{*}|\omega )$, assuming independent and Gaussian distributed measurement errors:

(47) $$p\left(y_{*}|\omega \right)={\left(2\pi \right)}^{-N_{*}/2}{\left|R\right|}^{-\frac{1}{2}}\exp\left[-\frac{1}{2}{\left(y_{*}-y_{k}\left(\omega ,t\right)\right)}^{T}R^{-1}\left(y_{*}-y_{k}\left(\omega ,t\right)\right)\right]\;$$

where R is the diagonal (co)variance matrix of size $N_{*}\times N_{*}$ ($N_{*}$ refers to the length of the observation data set) of measurement error ϵ. In our test case, we consider $N_{*}=10$ and a standard deviation of measurement error $\sigma_{\epsilon }=2$.

### 5.2. Bayesian Model Selection

**Figure 4:**
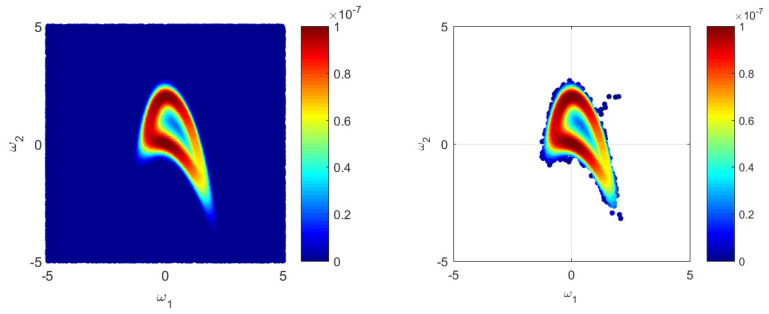
Likelihood values during the Bayesian updating via Monte Carlo (left plot) and Markov chain Monte Carlo (right plot) in a 2D reduction of the 10D problem.

We will use Monte Carlo sampling [48] with sample size $N_{\mathrm{prior}}=10^{6}$ to produce the MC-based reference solution ${\mathrm{BME}}^{\mathrm{Ref}}$ for the test scenario introduced in Section 5.1. Figure 4 illustrates how MC and MCMC approaches cover the parameter space. Here, in Figure 4 only, we reduced the 10D problem (46) to a 2D problem for illustrative purposes considering that there are only two parameters, i.e., $\omega_{i}=0$ for $i=\bar{3,10}$. All further computations presented here use the full 10D setup of the problem (46) from Section 5.1. The left plot in Figure 4 illustrates how the evaluated likelihood values cover the 2D parameter space using the MC approach. The right plot of Figure 4 illustrates the likelihood function as 2D sampled via Metropolis-Hastings-type MCMC algorithms [61] with the same sample size of $N_{\mathrm{post}}=10^{5}$. It is easy to see that the MCMC algorithm captures non-Gaussian aspects very effectively and, after a short burn-in phase (a few separate points in the upper right quadrant), places the samples in a high probability region. Other versions of MCMC could be used in a similar manner. To mitigate the bias resulting from the correlated nature of the samples, the usage of samples with a specified lag could be considered. Figure 4 reflects perfectly a typical application case of MCMC techniques, where only a posterior sample is available from MCMC in comparison to plain (prior-type) MC approaches. Due to the nature of appropriate MCMC techniques, a reliable posterior sample can be obtained at low computational costs in comparison to the plain MC approach. However, such an obvious advantage of MCMC poses a difficulty once a prior-based quantity of interest such as BME should be estimated.

To test our BME estimates from Section 3, we will compute a BME value from Equation (17) using the available MC sample, and compare it to the posterior-based estimates from Section 3 using the available MCMC sample. Figure 1 illustrates the performance of posterior-based ${\mathrm{BME}}_{\mathrm{post}}$ estimates against the prior-based ${\mathrm{BME}}_{\mathrm{prior}}$ estimate with respect to the sample size (MC or MCMC) relative to the reference value ${\mathrm{BME}}^{\mathrm{Ref}}$: ${\mathrm{BME}}_{\mathrm{post}}^{\mathrm{HM}}$ is the harmonic mean estimate [19], ${\mathrm{BME}}_{\mathrm{post}}^{\mathrm{MAP}}$ is the maximum a posteriori estimate from Equation (20), ${\mathrm{BME}}_{\mathrm{post}}^{\mathrm{CHIB}}$ is the Chib’s estimate from Equation (21), ${\mathrm{BME}}_{\mathrm{post}}^{\mathrm{AIC}}$ is the AIC-based estimate from Equation (24), ${\mathrm{BME}}_{\mathrm{post}}^{\mathrm{AICc}}$ is the AICc-based estimate from Equation (26), ${\mathrm{BME}}_{\mathrm{post}}^{\mathrm{KIC}}$ is the KIC estimate from Equation (30), ${\mathrm{BME}}_{\mathrm{post}}^{\mathrm{KICr}}$ is the KICr estimate from Equation (32), ${\mathrm{BME}}_{\mathrm{post}}^{\mathrm{BIC}}$ is the BIC estimate from Equation (34), ${\mathrm{BME}}_{\mathrm{post}}^{\mathrm{PDE}}$ is the posterior density estimate from Equation (19), ${\mathrm{BME}}_{\mathrm{post}}^{\mathrm{MG}}$ is the multivariate Gaussian posterior estimate from Equation (28) and ${\mathrm{BME}}_{\mathrm{post}}^{\mathrm{GD}}$ is the Gelfand and Dey posterior estimate from Equation (35). Due to normalization with ${\mathrm{BME}}^{\mathrm{Ref}}$, the ideal values is $\mathrm{BME}/{\mathrm{BME}}^{\mathrm{Ref}}$ = 1.

Figure 1 illustrates that the test scenario introduced in Section 5.1 is very challenging for most approximates. Our results confirm that the harmonic mean estimate performs poorly and suffers from large bias. The AIC-based and AICc-based estimates suffer from parameter dimensionality and their strong non-linearity. A similar situation could be observed for the BIC approximate. Though the maximum a posteriori estimate and Chib’s approximation demonstrate very similar results and a slightly better performance, this observation should not be generalized, as both approaches are very simplified estimates relying on the maximum a posteriori approximation. The BME estimate based on the KIC demonstrates a not satisfactory performance due to the fact that it does not act at the proper BME scale. The re-scaled KICr information criterion mitigates that problem and shows superior results. However, the KICr-based estimate includes unnecessary simplifications of the cross entropy $\hat{H}\left[p\left(\omega |y_{*}\right),p\left(y_{*}|\omega \right)\right]$ and the cross entropy $H\left[p\left(\omega |y_{*}\right),p\left(\omega \right)\right]$ using the maximum a posteriori estimate.

The last simplification is avoided by the posterior density estimate, the multivariate Gaussian posterior estimate and the Gelfand Dey approach. The performance of the posterior density estimate ${\mathrm{BME}}_{\mathrm{post}}^{\mathrm{PDE}}$ strongly depends on the related assumptions and problem dimensionality. The current 10D test scenario illustrates that the density estimator suffers from dimensionality, wchich often could be crucial for the approximation quality. We included the posterior density estimate only for demonstrational purposes, because this approach seems to be inefficient for high-dimensional problems. Figure 1 confirms the anticipations from Section 3, demonstrating a very acceptable performance for the multivariate Gaussian posterior estimate ${\mathrm{BME}}_{\mathrm{post}}^{\mathrm{MG}}$. The Gelfand and Dey approach provides slightly inferior results. Nevertheless, it includes assumptions similar to the multivariate Gaussian estimate. Both approaches have the potential to capture the posterior better than other estimates discussed in the paper. However, once the posterior is extremely non-Gaussian, both mentioned approximates could be less powerful by their definitions. In that situation Equation (18) explicitly shows that the main computational efforts should be focused on the estimation of the third term responsible for the posterior information entropy $H\left[p(\omega |y_{*})\right]$. Overall, the multivariate Gaussian posterior estimate introduced in Equation (28) avoids unreasonable simplifications and leads to a superior BME estimate from MCMC-based posterior samples.

### 5.3. Information Entropy and Bayesian Experimental Design

We will estimate the information entropy during Bayesian updating using $H\left[p(\omega |y_{*})\right]$. Similar to Section 5.2, we will compute a reference value $H^{\mathrm{Ref}}\left[p(\omega |y_{*})\right]$ using plain MC techniques with sample size $N_{\mathrm{prior}}=10^{6}$ according to Equation (38), avoiding multidimensional density estimation and integration. To estimate the information entropy via the MCMC-based posterior sample, we will employ Equation (39) using the various BME estimates introduced in Section 3 and illustrated in Section 5.2. Figure 2 compares the performance of these posterior-based estimates for $H_{\mathrm{post}}$ against the prior-based value $H_{\mathrm{prior}}$ relative to the reference value $H^{\mathrm{Ref}}\left[p(\omega |y_{*})\right]$. Again, the ideal value is unity. It easy to see that the information entropy estimated via the multivariate Gaussian posterior from Equation (28) and via the Gelfand and Dey estimate from Equation (35) show the most suitable performance. Moreover, the KICr-based estimate shows results similar to the multivariate Gaussian estimate due to the definition in Equation (32). Thus, for posterior-based model selection based on the information entropy $H\left[p(\omega |y_{*})\right]$, we suggest to employ the multivariate Gaussian posterior estimate or the Gelfand and Dey approach.

Next, we look at the Bayesian experimental design where the utility of experiment outcome in terms of the relative entropy $D_{\mathrm{KL}}\left[p\left(\omega |y_{*}\right),p\left(\omega \right)\right]$ should be computed, and compare our estimates in Equation (43) to the MC-based reference solution. Figure 3 shows the convergence of our estimates relative to the MC reference value $D_{\mathrm{KL}}^{\mathrm{Ref}}\left[p\left(\omega |y_{*}\right),p\left(\omega \right)\right]$ obtained from Equation (42) with sample size $N_{\mathrm{prior}}=10^{6}$. Both prior and posterior-based estimates of the relative entropy $D_{\mathrm{KL}}\left[p\left(\omega |y_{*}\right),p\left(\omega \right)\right]$ avoid the multidimensional integral using the link between information theory and Bayesian inference offered in Section 2. The multivariate Gaussian approximation and the Gelfand-Dey approach provide very reasonable estimates with least assumptions and seem to be very useful for practical application.

Overall, the current section, Section 5.3, illustrates estimates of the information entropy during Bayesian updating and for experimental design. The prior-based estimates avoid unnecessary computation of multidimensional integrals and include no additional assumptions. The posterior-based estimates avoid as well the high-dimensional integrals, however they include at least one additional assumption. By definitions, the multivariate Gaussian posterior estimate and the Gelfand and Dey estimate for BME, $H\left[p(\omega |y_{*})\right]$ and $D_{\mathrm{KL}}\left[p\left(\omega |y_{*}\right),p\left(\omega \right)\right]$ include least assumptions among all approximations discussed in Section 3 and hence seem to be the most suitable one for practical applications.

## 6. Summary and Conclusions

The current paper shows the link between Bayesian inference and information theory. We align Bayesian model evidence (BME) with relative entropy and with cross entropy in order to simplify computations for model selection, assessment of information entropy and experimental design. First, we demonstrate how Bayesian model selection can profit from information theory to estimate BME via posterior-based techniques such as MCMC. We show that MCMC-based Bayesian model selection could be achieved using several assumptions such as a maximum a posteriori estimate or a multivariate Gaussian posterior. Additionally, we link BME value to the AIC and AICc information criteria and provide a new re-scaling of the KIC criterion. Second, we demonstrate how relative entropy could profit from BME to assess information entropy during Bayesian updating and to assess the utility of experimental outcomes for Bayesian experimental design. The current paper emphasizes that relative entropy could be computed avoiding unnecessary multidimensional integration for both prior and posterior-based techniques. Prior-based approaches do not require any assumptions for estimating relative entropy. Estimating relative entropy using posterior sampling approaches requires at least one assumption.

We illustrate the performance of the introduced estimates for BME, information entropy and experiment utility using a numerical reference solution for a very simple example. The well-known harmonic mean estimate for BME demonstrates weak performance and provides very unreliable results. The maximum a posteriori, Chib’s estimate, AIC-based estimate, AICc-based estimate and BIC estimate seem to be very simplified and can offer first rough guesses only. An estimate based on the KIC information criteria demonstrates unsatisfactory performance because it does not act at the proper BME scale. Its re-scaling KICr mitigates that problem and shows superior results. However, KICr-based estimates include unnecessary simplifications of involved cross entropies using the maximum a posteriori estimate. The multivariate Gaussian posterior estimate avoids unreasonable simplifications and includes least assumptions for estimating BME, information entropy and experiment utility for posterior-based techniques. The Gelfand and Dey approach provides slightly inferior results. However, it includes assumptions similar to the multivariate Gaussian estimate and, hence, both approaches have a potential to capture the posterior better than other estimates discussed in the paper.

Overall, we conclude that the introduced relation of Bayesian inference to information theory could be very helpful for applied tasks where Bayesian model evidence, information entropy and experimental utility should be assessed via prior-based or posterior-based techniques. 

## Figures and Tables

**Table 1:** Summary of assumptions behind estimates.

Estimate and Equation Number	Non-Normalized Cross Entropy $\hat{H}\left[p\left(\omega |y_{*}\right),p\left(y_{*}|\omega \right)\right]$	Cross Entropy $H\left[p\left(\omega |y_{*}\right),p\left(\omega \right)\right]$	Information Entropy $H\left[p(\omega |y_{*})\right]$
PDE estimate (19)	No assumptions	No assumptions	Kernel density estimation
MAP estimate (20)	No assumptions	No assumptions	MAP point estimates
Chib estimate (21)	MAP point value	MAP point value	MAP point estimates
AIC estimate (24)	No assumptions	No assumptions	AIC estimates
AICc estimate (26)	No assumptions	No assumptions	AICc estimates
MG estimate (28)	No assumptions	No assumptions	MG estimates
KIC estimate (30)	MAP point estimates	MAP point estimates	KIC estimates
KICr estimate (32)	MAP point estimates	MAP point estimates	MG estimates
BIC estimate (34)	MAP point estimates	Asymptotical limit for growing data size	
GD estimates * (35)	No assumptions	No assumptions	MG estimates

* Remark: GD estimates relies on similar assumptions, but have different representation.
